# Pre-Vaccine Positivity of SARS-CoV-2 Antibodies in Alberta, Canada during the First Two Waves of the COVID-19 Pandemic

**DOI:** 10.1128/spectrum.00291-21

**Published:** 2021-08-18

**Authors:** Carmen L. Charlton, Leonard T. Nguyen, Ashley Bailey, Jayne Fenton, Sabrina S. Plitt, Carol Marohn, Cheryl Lau, Deena Hinshaw, Christie Lutsiak, Kimberley Simmonds, Jamil N. Kanji, Nathan Zelyas, Nelson Lee, Michael Mengel, Graham Tipples

**Affiliations:** a Public Health Laboratory, Alberta Precision Laboratories, Alberta, Canada; b Department of Laboratory Medicine and Pathology, University of Alberta, Edmonton, Alberta, Canada; c Li Ka Shing Institute of Virology, University of Alberta, Edmonton, Alberta, Canada; d Alberta Precision Laboratories, Edmonton, Alberta, Canada; e Centre for Communicable Diseases and Infection Control, Public Health Agency of Canada (PHAC), Ottawa, Ontario, Canada; f Alberta Ministry of Health, Edmonton, Alberta, Canada; g Department of Community Health Sciences, University of Calgary, Calgary, Alberta, Canada; h Department of Medicine, Faculty of Medicine and Dentistry, University of Alberta, Edmonton, Alberta, Canada; i Division of Infectious Diseases, Department of Medicine, Cumming School of Medicine, University of Calgary, Calgary, Alberta, Canada; j Division of Infectious Diseases, Department of Medicine, Faculty of Medicine and Dentistry, University of Alberta, Edmonton, Alberta, Canada; k Department of Medical Microbiology and Immunology, University of Alberta, Edmonton, Alberta, Canada; Children’s Hospital Los Angeles, University of Southern California

**Keywords:** SARS-CoV-2, antibody, immunology, nucleocapsid protein, serology, seroprevalence, spike protein

## Abstract

We systematically evaluated SARS-CoV-2 IgG positivity in a provincial cohort to understand the local epidemiology of COVID-19 and support evidence-based public health decisions. Residual blood samples were collected for serology testing over 5-day periods monthly from June 2020 to January 2021 from six clinical laboratories across the province of Alberta, Canada. A total of 93,993 individual patient samples were tested with a SARS-CoV-2 nucleocapsid antibody assay with positives confirmed using a spike antibody assay. Population-adjusted SARS-CoV-2 IgG seropositivity was 0.92% (95% confidence interval [CI]: 0.91 to 0.93%) shortly after the first COVID-19 wave in June 2020, increasing to 4.63% (95% CI: 4.61 to 4.65%) amid the second wave in January 2021. There was no significant difference in seropositivity between males and females (1.39% versus 1.27%; *P = *0.11). Ages with highest seropositivity were 0 to 9 years (2.71%, 95% CI: 1.64 to 3.78%) followed by 20 to 29 years (1.58%, 95% CI: 1.12 to 2.04%), with the lowest rates seen in those aged 70 to 79 (0.79%, 95% CI: 0.65 to 0.93%) and >80 (0.78%, 95% CI: 0.60 to 0.97%). Compared to the seronegative group, seropositive patients inhabited geographic areas with lower household income ($87,500 versus $97,500; *P < *0.001), larger household sizes, and higher proportions of people with education levels of secondary school or lower, as well as immigrants and visible minority groups (all *P < *0.05). A total of 53.7% of seropositive individuals were potentially undetected cases with no prior positive COVID-19 nucleic acid test (NAAT). Antibodies were detectable in some patients up to 9 months post positive NAAT result. This seroprevalence study will continue to inform public health decisions by identifying at-risk demographics and geographical areas.

**IMPORTANCE** Using SARS-CoV-2 serology testing, we assessed the proportion of people in Alberta, Canada (population 4.4 million) positive for COVID-19 antibodies, indicating previous infection, during the first two waves of the COVID-19 pandemic (prior to vaccination programs). Linking these results with sociodemographic population data provides valuable information as to which groups of the population are more likely to have been infected with the SARS-CoV-2 virus to help facilitate public health decision-making and interventions. We also compared seropositivity data with previous COVID-19 molecular testing results. Absence of antibody and molecular testing were highly correlated (95% negative concordance). Positive antibody correlation with a previous positive molecular test was low, suggesting the possibility of mild/asymptomatic infection or other reasons leading individuals from seeking medical attention. Our data highlight that the true estimate of population prevalence of COVID-19 is likely best informed by combining data from both serology and molecular testing.

## INTRODUCTION

Shortly after the emergence of SARS-CoV-2 in Wuhan province, COVID-19 was officially declared a pandemic by the World Health Organization on 11 March 2020. The first documented case of SARS-CoV-2 infection in Canada was recorded on 25 January 2020, in a passenger arriving in Toronto, Ontario indirectly from Wuhan (1). In the Western Canadian province of Alberta, the first official case was diagnosed by *post hoc* testing to have occurred on 24 February 2020 in an individual arriving following travel to the Western United States (2). Since that time, Alberta has performed over 4.2 million molecular tests for SARS-CoV-2 (as of 3 May 2021) (3). High testing rates with rapid turnaround allow for timely contact tracing of all positive cases. This improves the effectiveness of public health interventions to identify individuals who may have been exposed to the virus and to isolate them to prevent spread within the community.

Two waves of infection have been experienced in Alberta; the first wave had peak testing positivity rates of nearly 6% and occurred between March and May 2020 (4). The second wave started in October 2020, reaching a peak 1-day positivity of 10% in mid-December 2020. Despite the high testing volume in Alberta, some proportion of cases would have been undiagnosed or asymptomatic. Serology testing in this population provides us with additional information on the level of transmission and the concordance of serology testing within our population after and during COVID-19 waves in the province.

To further support decision-making by our public health teams and determine the presence of unidentified populations with higher prevalence of COVID-19, residual clinical blood samples from across Alberta were collected over 5-day periods each month. COVID-19 seropositivity rates were compared between June, July, August, September, October, November, and December 2020 and January 2021 patients. We assessed samples for COVID-19 seropositivity and stratified them by age, sex, and geographic location to better understand the progression of SARS-CoV-2 infection in the province. These results were additionally stratified by individuals who were previously tested by molecular tests to assess the concordance between serology and molecular testing.

## RESULTS

### Patient demographics.

Here, we present a snapshot of SARS-CoV-2 antibody positivity in the Alberta population from June 2020 to January 2021 prior to general population vaccine implementation. Serology testing was performed on 104,723 residual clinical chemistry samples. After duplicate specimens and those captured outside the established time periods were excluded, 93,993 samples from separate patient encounters were kept for analysis. Over the eight monthly surveyed periods, the median patient age was 58 years (interquartile range [IQR]: 40 to 70), and the proportion of females was 56.2%. A total of 50.8% of the surveyed patients resided in the two largest cities of Alberta: 25,479 in Calgary (27.1%) and 22,260 in Edmonton (23.7%). A total of 35.0% resided in the remaining urban municipalities of Alberta (*n *= 32,905), and 12.9% were from rural areas (*n *= 12,107). A total of 0.94% of the surveyed patients were under 10 years of age (*n *= 885).

### Epidemiology of provincial SARS-CoV-2 infections.

**FIG 1:**
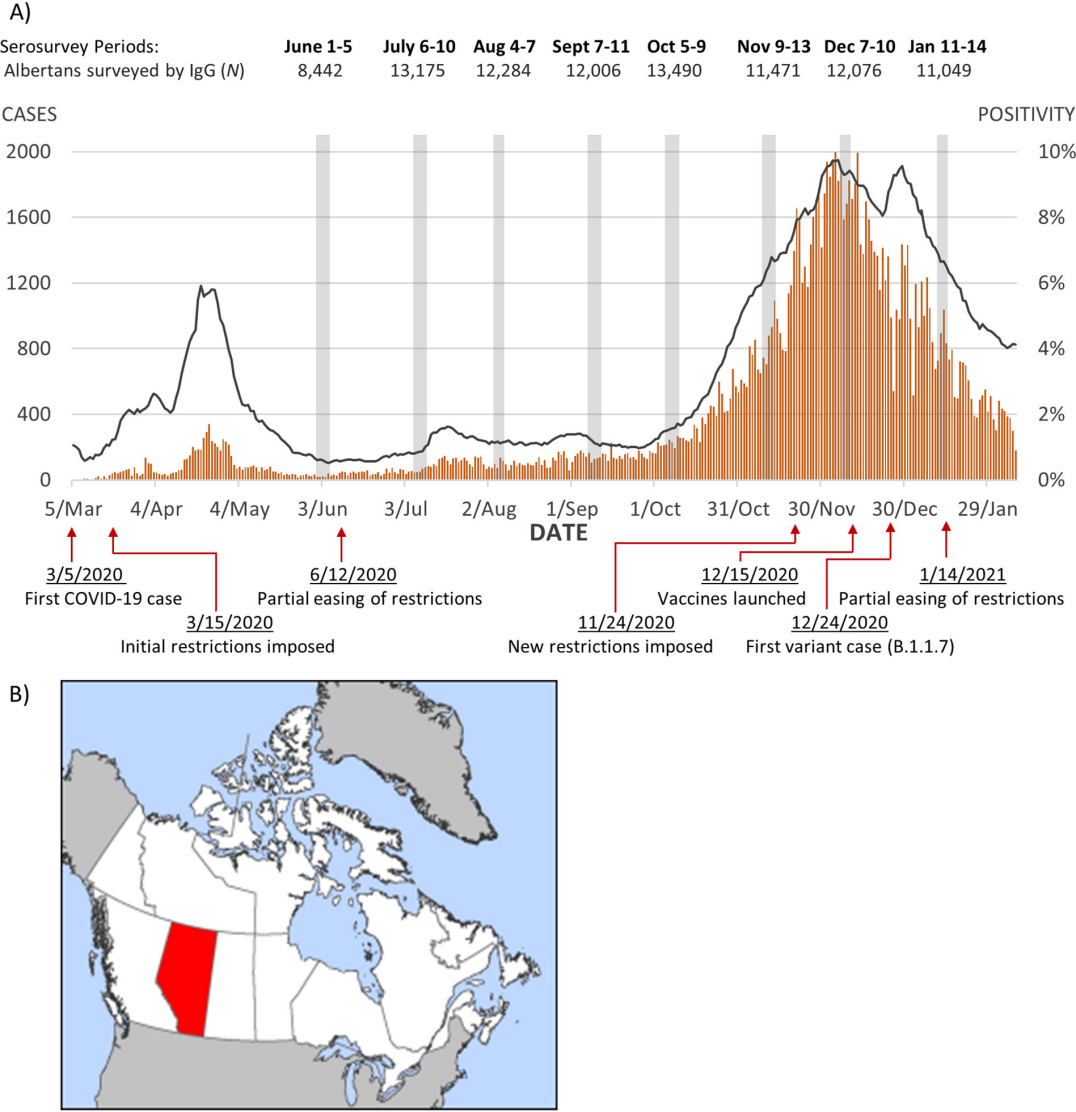
(A) Daily new cases and positivity rates of COVID-19 NAT results verified in Alberta with monthly serosurvey periods and major pandemic-related events. (B) Location of Alberta in Canada. Map taken from the public domain (https://en.m.wikipedia.org/wiki/File:Alberta-map.png).

In Alberta, the first COVID-19 wave peaked around 24 April 2020 with a 7-day average nucleic acid test (NAAT) positivity of 5.80% (Fig. 1). The first samples for this study were collected between 1 and 5 June 2020, approximately 6 weeks after the first wave. Daily case counts were low from June through September 2020, with NAAT positivity staying under 2% until 6 October 2020. Overall seropositivity over the time period studied was 1.33% (95% CI 1.25 to 1.40) (crude) and 1.75% (95% CI 1.74 to 1.76) (adjusted). Seroprevalence peaked in January 2021 (crude 3.63%; adjusted 4.63%) (Table 1).

**TABLE 1:** Summary characteristics and prevalence rates for each serosurvey period (June, 2020–January, 2021)^*a*^

Mo	Albertans tested (*n*)	Positive IgG (*n*_POS_)	% Female (IgG pos)	Median age (IgG pos)	% Seroprevalence, crude (95% CI)	% Seroprevalence, adjusted^*b*^ (95% CI)
June 2020	8,442	78	50.0	48	0.92 (0.72–1.13)	0.92 (0.91–0.93)
July 2020	13,175	68	50.0	41.5	0.52 (0.39–0.64)	0.86 (0.85–0.87)
August 2020	12,284	92	56.5	51	0.75 (0.60–0.90)	1.24 (1.23–1.25)
September 2020	12,006	115	56.5	58	0.96 (0.78–1.13)	1.02 (1.00–1.02)
October 2020	13,490	119	61.3	52	0.88 (0.72–1.04)	1.16 (1.15–1.17)
November 2020	11,471	132	48.5	48	1.15 (0.96–1.35)	1.61 (1.60–1.62)
December 2020	12,076	241	49.0	46	2.00 (1.76–2.26)	2.47 (2.46–2.49)
January 2021	11,049	400	57.0	48	3.63 (3.28–3.98)	4.63 (4.61–4.65)
All mo	93,993	1,245	54.1	49	1.33 (1.25–1.40)	1.75 (1.74–1.76)

a mo, month(s).

*^b^*Standardized by sex and age group distribution in Alberta (29).

COVID-19 cases began to rise in October, reaching a maximum 7-day average rate of 9.74% on 6 December 2020 and declining from this second wave in January 2021 (Fig. 1). The seropositivity rates increased in these months with age and sex standardized rates of 1.61% in November (95% CI: 1.60 to 1.62), 2.47% in December (95% CI: 2.46 to 2.49), and 4.63% in January 2021 (95% CI: 4.61 to 4.65) (Table 1).

### Antibody positivity by sex and age.

**FIG 2:**
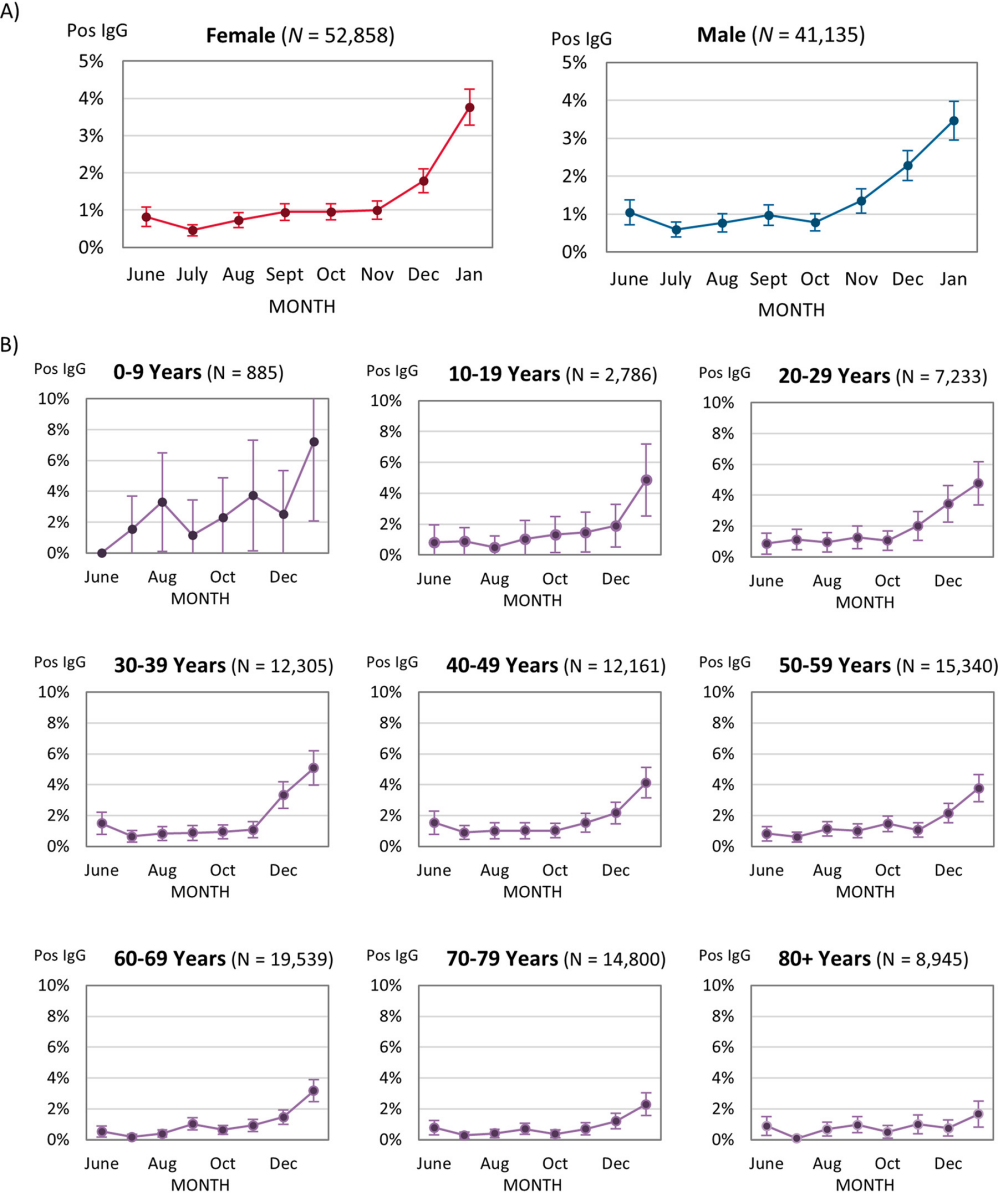
IgG positivity across the serosurvey periods by (A) sex and (B) age group, with 95% CI error bars.

There were no significant differences in seropositivity by sex over the combined survey periods (*P = *0.11) (Table 2) or in most months, except for December, when seroprevalence among males was 2.27% (95% CI: 1.87 to 2.67%) and that among females was 1.77% (95% CI: 1.46 to 2.09%), with a *P *value of 0.05 (Fig. 2A; Table S2).

**TABLE 2:** Overall COVID-19 seroprevalence of Alberta by sex, age groups, and geography, June 2020 to January 2021 (*N *= 93,993)

Demographic	Survey size (*N*)	IgG positive count (*n*_POS_)	% IgG positive (95% CI)
Sex			
Male	41,135	572	1.39 (1.28–1.50)
Female	52,858	673	1.27 (1.18–1.37)
Unknown	2	0	0
Age group (y)			
0–9	885	24	2.71 (1.64–3.78)
10–19	2,786	44	1.58 (1.12–2.04)
20–29	7,233	140	1.94 (1.62–2.25)
30–39	12,305	217	1.76 (1.53–2.00)
40–49	12,161	202	1.66 (1.43–1.89)
50–59	15,340	231	1.51 (1.31–1.70)
60–69	19,539	200	1.02 (0.88–1.16)
70 to 79	14,800	117	0.79 (0.65–0.93)
≥80	8,945	70	0.78 (0.60–0.97)
City/area			
Calgary	25,479	310	1.22 (1.08–1.35)
Edmonton	22,260	361	1.62 (1.46–1.79)
Red Deer	2,471	16	0.65 (0.33–0.96)
Lethbridge	4,102	20	0.49 (0.27–0.70)
Medicine Hat	4,413	11	0.25 (0.10–0.40)
Grande Prairie	2,619	12	0.46 (0.20–0.72)
Brooks	1,170	124	10.6 (8.83–12.36)
Rural	12,107	247	2.04 (1.79–2.29)
Alberta, other	18,130	120	0.66 (0.54–0.78)

Overall, the median age of patients positive for SARS-CoV-2 antibodies was 49 years (IQR: 35 to 63) (Fig. 2; Table 1; Table S2). The youngest median age for seropositive patients occurred in July (41.5 years, IQR: 32 to 55.8), and the oldest median age occurred in September (58 years, IQR: 51.5 to 69). Over the entire survey period, children 0 to 9 years old had the highest seropositivity (2.71%, 95% CI: 1.64 to 3.78%), and this seropositivity was significantly higher than that of all other age groups except for the 20- to 29-year-olds, who had the second highest overall seropositivity (1.94%, 95% CI: 1.62 to 2.25%). There were 202/885 (22.8%) children aged ≤2 years of age, of which 5/202 (2.5%) were seropositive.

During the second wave from November 2020 to January 2021, seropositivity increased significantly for all age groups except among 0- to 9- and ≥80-year-olds. In January, the highest seropositivity was seen among patients 0 to 9 years old (7.22%, 95% CI: 2.07 to 12.37%) followed by those 30 to 39 years old (5.07%, 95% CI: 3.95 to 6.19%), while the seropositivity for those ≥80 years old was lowest at 1.67% (95% CI: 0.83 to 2.50%).

### Antibody positivity by geography and socioeconomic demographics.

Compared to that of most other cities, Calgary had a higher seropositivity in June (3.53%, 95% CI: 2.20 to 4.86%) (Fig. S1; Table S3), which aligns with the city’s known high infection rate in Spring 2020. The seropositivity for Calgary decreased to below provincial average until September onwards, when it rose again in conjunction with the second wave. In Edmonton, seropositivity was below or near the provincial average until October, after which it increased to 5.95% (95% CI: 5.01 to 6.89%) in January compared to 3.63% (95% CI: 3.28 to 3.98%) for the province. The midsized cities of Red Deer, Lethbridge, Medicine Hat, and Grande Prairie all had seropositivities below 1% except for the increase in January to 3.42% (95% CI: 1.43 to 5.40%) for Red Deer. The small municipality of Brooks had a very high seropositivity rate of 19.8% (95% CI: 11.4 to 28.2%) in June, largely due to an outbreak at a large meat-packing plant 2 months earlier. Although the seropositivity in Brooks decreased over time, it consistently remained the highest in the province with a mean seroprevalence of 10.6% (95% CI: 8.83 to 12.36%) over the 8 study months compared to 1.21% (95% CI: 1.14 to 1.28%) for the rest of the province (Table 2). From August to January, seropositivity in rural areas was consistently above the provincial average and higher than that in most urban municipalities.

Overall, patients who were seropositive came from neighborhoods with median household incomes lower compared to those of seronegative individuals ($87,500, 95% CI: $85,600 to $89,500 compared to $97,500, 95% CI: $97,300 to $97,800, respectively; *P < *0.001) (Table S4). Dissemination areas (DAs) for seropositive patients had significantly lower marriage rates, larger household sizes, and higher proportions with education level at or below high school (all *P < *0.001) (Table S4). Among DAs for seropositive patients, the proportions of immigrants (27.2%, 95% CI: 26.3 to 28.2%) and visible minorities (30.9%, 95% CI: 29.5 to 32.4%) were significantly higher than those for seronegative patients (21.2%, 95% CI: 21.1 to 21.3% and 22.9%, 95% CI: 22.7 to 23.0%, respectively; both *P < *0.001).

### Longevity of nucleocapsid antibodies.

**FIG 3:**
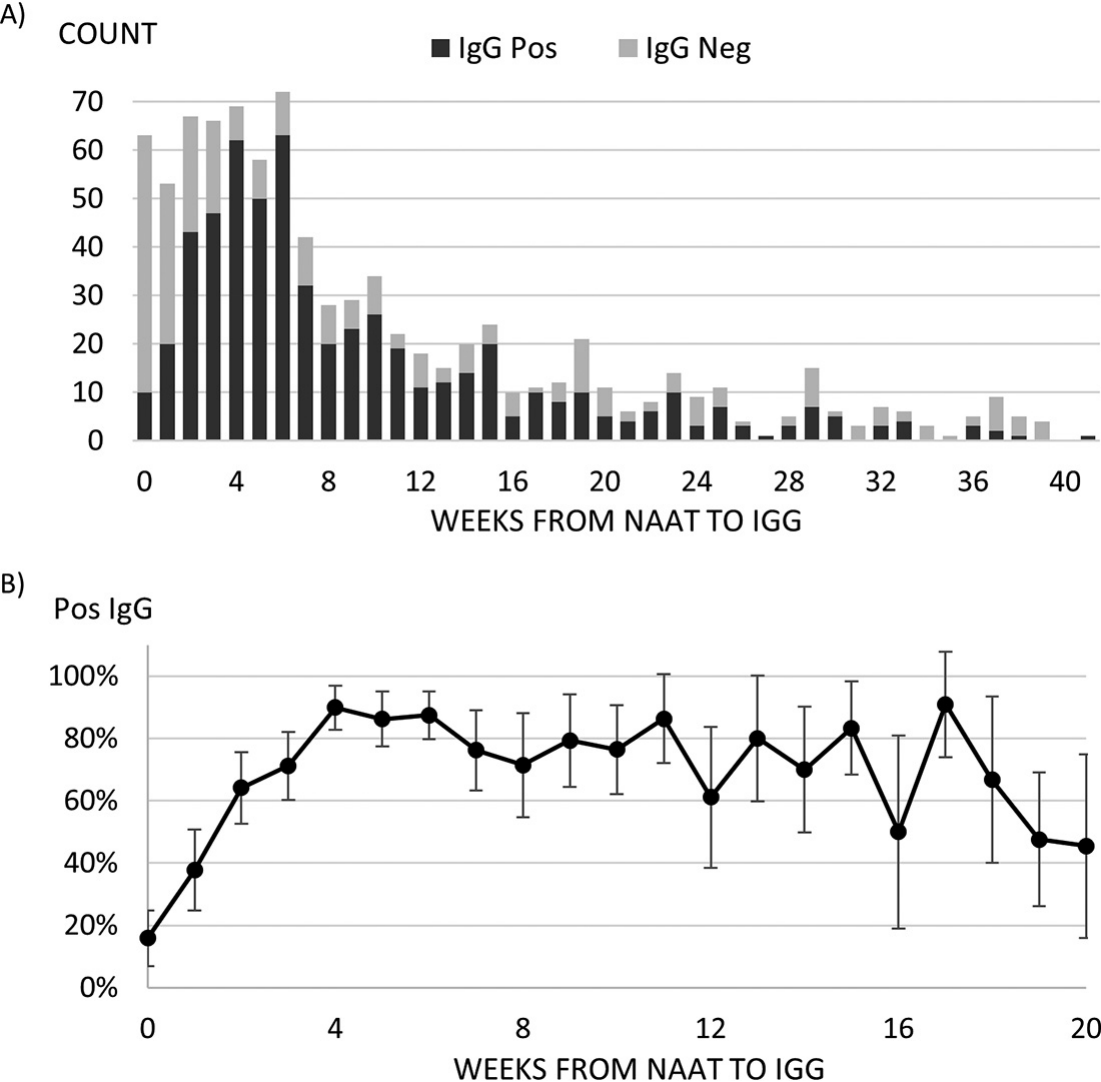
(A) Counts of IgG status and (B) positivity rates for patients with positive NAAT results by number of weeks from NAAT to serology test (*n *= 867). The number of patients with greater than 20 weeks from positive NAAT result to serology test.

Detection of SARS-CoV-2 nucleocapsid antibodies in patients who tested positive by NAAT increased in the first month following NAAT, reaching a maximum seropositivity of 89.9% (95% CI: 82.7 to 97.0%) on week 4 after positive NAAT (Fig. 3). After week 6, antibody detection decreased to 50.0% (95% CI: 19.0 to 81.0) on week 16. After week 20, large fluctuations in seropositivity were observed with large uncertainties due to sample sizes smaller than 10. However, a positive serology result was observed in a patient on week 41 (9 to 10 months) after the patient’s last recorded positive NAAT result.

If the criteria for seropositivity were adjusted by omitting the spike protein confirmation and lowering the nucleocapsid antibody instrument cutoff from 1.4 to 0.7 (5–7), the sensitivity of SARS-CoV-2 antibody testing to detect previously positive NAAT cases increased from 66.1% to 85.0% (Table S5). This same adjustment for positivity criteria did not have a great effect on concordance (97.9% versus 99.3% with the original 1.4 antibody with spike protein confirmation criteria). While the number of potential false-negative samples (NAAT-positive but serology-negative) decreased from 294 to 130 by dropping the cutoff, the number of potential false positives (NAAT-negative but serology-positive) increased from 179 to 539. Given the higher agreement of the 0.7 cutoff with NAAT testing, applying this looser positivity threshold would increase the population-adjusted positivity of Alberta in January 2021 from 4.63% (95% CI: 4.61 to 4.65%) to 7.24% (95% CI: 7.21 to 7.26%) (Fig. S2).

### Correlation between NAAT and antibody testing.

Overall, 71.0% of surveyed patients (*n *= 66,171) were not previously tested by NAAT in the province (Table S6). Out of 1,237 patients testing positive by serology, 664 (53.7%) were undetected COVID-19 cases by having either no previous NAAT testing (485, 39.2%) or only negative NAAT results (179, 14.4%). Conversely, 294/867 (33.9%) of NAAT-positive patients were negative by serology.

Comparison of SARS-CoV-2 IgG positive individuals who were previously NAAT positive and those who were either NAAT negative or had no NAAT testing performed demonstrates no difference between gender, age groups 10 to 79 years, and residence in a midsize urban center (*P > *0.05) (Table S6). Individuals aged 0 to 9 years and those living in rural areas were significantly more likely either to have not had a NAAT test or to have previously been NAAT negative prior to their positive IgG result (*P < *0.05). Conversely, those ≥80 years in age, living in major urban centers, and found to have a higher median household income were more likely to have had a documented positive NAAT result leading up to their IgG test date (*P < *0.05).

## DISCUSSION

From a public health perspective, it is important to understand where infections are occurring and if there are populations where unidentified transmission is taking place. Understanding which populations are most affected by SARS-CoV-2 provides an opportunity to target additional supports to reduce disease transmission. We have shown that individuals from geographic areas having lower education, lower income, and increased proportions of visible minorities are most likely to be positive for SARS-CoV-2 antibodies. These factors could be related to employment in industries that do not allow work from home options, for example taxi services or warehouse workers, or related to employment in areas that have already experienced outbreaks, such as the meat-packing plants in Brooks. While it does not evaluate mortality from COVID-19, this finding from Alberta is consistent with those from other locales worldwide (8–11).

Interestingly, after July 2020, pediatric patients (≤9 years of age) were found to have a modest positivity for SARS-CoV-2 antibodies (ranging from 1.2 to 7.3%) (Fig. 2). This is inconsistent with laboratory-confirmed NAAT positives for this age group, which had NAAT positivity rates similar to those of patients 10 to 59 years of age (12% positivity in ages 0 to 9 years, 13% positivity in ages 10 to 19 years, 15% in 20 to 29, 18% in 30 to 39, 14% in 40 to 49, and 12% in 50 to 59) (3). The elevated antibody level in those >9 years of age (without a corresponding increase in NAAT positives) could indicate that many of the infections in children are either asymptomatic or mild enough not to warrant a health care visit or laboratory testing. This is also supported by our finding that seropositive children in this age group were more likely to not have had any previous NAAT testing than have a positive NAAT test. However, the low number of patients tested monthly in this age group brings high uncertainty, and results may not be representative of the general population in this age group as children who had serology samples taken may have higher risk factors for COVID-19 infection than the general population.

In addition to determining population demographics of those with positive SARS-CoV-2 IgG, we examined the effectiveness of two-step or orthogonal serology testing in our population. Two approaches have been considered for surveillance testing, single screening assays and a two-step approach where positive screening assays are confirmed with a second assay (generally targeting a different antibody epitope). While orthogonal serology testing was initially recommended to increase the confidence in positive antibody results, and is currently used in most public health laboratories in Canada, its role in surveillance studies is now being questioned (12, 13). In cases where it is essential to ensure a sample is positive for SARS-CoV-2 antibodies, such as plasma enrichment, an orthogonal algorithm is still indicated; however, in cases where false positives are less of a concern and, as in the case for serosurveillance studies, false negatives should be minimized, orthogonal testing may not be the best approach. This is supported by our data where only 66.7% of samples that were positive by the architect nucleocapsid antibody (cutoff ≥1.4) were confirmed positive by the DiaSorin spike antibody assay. When we examined positivity rates for January, there was a significant difference between single assay and two-step (orthogonal) testing (4.63% positivity compared to 7.24% adjusted positivity, respectively) (Fig. S2). Similar to what we have observed, other groups have found that population screening using a lower cutoff value describes more accurately the NAAT positivity rate seen in the province (7). Likewise, we see a higher positivity rate of 4.2% if we use single assay detection at a cutoff of ≥0.7 for the month of December, which is more consistent with the approximately 5% NAAT positivity rate seen 2 to 3 weeks prior to serum collection (assuming antibodies are not detected until 2 to 3 weeks post NAAT-positive result) (14). As the prevalence, and likewise the positive predictive value, of previous SARS-CoV-2 infection increases in our population, the need for orthogonal testing will likely continue to decrease.

To further examine the relationship between NAAT and serology positivity, we linked previous NAAT testing results to serology IgG results. Using the orthogonal algorithm, we found that 0.7% of patients negative by NAAT were subsequently positive by IgG (Table S5). Some cross-reactivity to other respiratory viruses has been observed with commercial SARS-CoV-2 serology assays (14), which may explain, in part, the discordance between NAAT and antibody testing (particularly as many respiratory viruses have overlapping clinical symptom presentations). From our experience, we have observed the DiaSorin SARS-CoV-2 IgG assay display a false positive for someone who was SARS-CoV-2 negative and recovered from parainfluenza virus (14). From that study, while we did not observe any cross-reactivity of the Abbott Architect with a panel of well-characterized prepandemic sera, other IgG assays evaluated demonstrated cross-reactivity with human metapneumovirus, rhinovirus/enterovirus, and coronavirus 229E. This may contribute to the higher positivity potentially seen in children, but to what extent is not certain. However, a positive serology with a negative NAAT does not necessarily mean the serology was a false positive, as timing and quality of NAAT collection may contribute to a negative result (15). Additionally, previous studies examining SARS-CoV-2 and Middle East respiratory virus (MERS-CoV) NAAT detection showed false-positive rates ranging from 2% to 30% (average 8%) (16, 17). Overall, the discordance between the two methods is very low and within the range of the manufacturer’s published performance criteria.

Initial reports on antibody longevity suggested a rapid decline of the nucleocapsid and, to a lesser degree, spike antibodies after 2 to 3 months, while larger, more recent long-term studies have shown antibodies to last up to 4 or 6 months after infection (18–22). In our study, we detected nucleocapsid and spike antibodies against SARS-CoV-2 up to 36 weeks (8 months) post NAAT positive. While the numbers were small, we did not detect antibodies to nucleocapsid in samples collected at 37 or 38 weeks post NAAT-positive result. Only a very small proportion of individuals did not mount an antibody response following a positive PCR result (0.59%), suggesting that most individuals will mount a humoral response following natural infection. One limitation to this study is that a nucleocapsid antibody assay was used as the first screening test, and therefore antibody to spike is assessed only in samples positive for nucleocapsid antibody.

Our serology positivity results are similar to those of other published studies, including the Canadian Blood Services (CBS) screening study looking at over 5,000 Albertans and a more recent study examining the Canadian blood-donor population (23, 24). One major difference observed was in the Calgary region. Using the same screening assay, the CBS study observed 0.5% positivity, while we observed 3.4% positivity in the same region for the month of June. This may represent sampling biases between the studies, as our study represents patients seeking health care while the CBS study was performed on healthy Canadians, or it may represent a sampling bias in our study due to the comparatively smaller number of samples obtained from the Calgary region during this time. However, the daily NAAT positivity level in the 2 to 3 weeks preceding the June serology sampling time period was 3.5% on average, suggesting that the positivity in the population may be higher than that observed in the blood-donor population group.

One limitation to this study is the use of residual samples, which are not completely representative of the population. Patients who have accessed health care and who required analytical chemistry testing were included in this study, but individuals of good health who did not access health care during the sample collection periods were not included. Therefore, positivity rather than true population prevalence has been calculated. Additionally, there is a sampling bias for large urban centers, as access to care in these centers has more entry points than that in more rural regions. While we attempted to mitigate this disparity by increasing the collection time for samples from more rural sites, this bias cannot be totally ignored. Additionally, we have used census data as a proxy for individual data, which may have led to misclassification of these sociodemographic variables for individual patients, and it is also possible that the sociodemographic data shifted for regions between the time of the 2016 census to our analysis in 2020. However, this use of census data should identify general trends, and our results of COVID seroprevalence being higher among visible minorities group and those of lower socioeconomic status have been reported previously (25, 26).

Here, we present a snapshot of SARS-CoV-2 antibody positivity in Alberta, Canada prior to vaccination of the general population covering both the first and the second waves of infection. We show that antibodies against SARS-CoV-2 are detectable up to 8 months post NAAT-positive result for both nucleocapsid and spike proteins, and while this has not been correlated with immunity from subsequent infection, it is reassuring that antibodies may be longer lasting than initially thought. Furthermore, we show that a single serological assay (compared to an orthogonal algorithm) has improved correlation to provincial NAAT positivity rates in this large-scale population-based study and suggest that both the positive predictive value and the need to mitigate false positives be considered when implementing serological algorithms in the clinical laboratory.

## MATERIALS AND METHODS

### Ethics statement.

Ethics approval for this research was received from the University of Alberta Health Research Ethics Board, approval number Pro00101916.

### Sample collection.

From June 2020 to January 2021, residual blood specimens (serum, plasma, and heparinized plasma) were gathered monthly from clinical testing laboratories across Alberta and sent to the Public Health Laboratory (ProvLab) for SARS-CoV-2 serology testing. These samples were collected by phlebotomy from community patients for test requests unrelated to COVID-19 serology. For each monthly period, the larger laboratories in the two major Alberta cities, Calgary and Edmonton, contributed 1 day’s worth of blood samples (approximately 4,000 to 5,000 samples from each site). The monthly contributions from the laboratories at the Regional Hospitals in smaller cities of Grande Prairie, Red Deer, Lethbridge, Brooks, and Medicine Hat consisted of 4 to 7 consecutive days’ worth of blood samples (approximately 500 to 1,000 samples from each site). The wider time windows for these laboratories, which also serve as testing hubs for their surrounding rural areas, were chosen to increase representation from around the province.

### SARS-CoV-2 nucleic acid testing.

As part of its public health strategy, Alberta offered SARS-CoV-2 molecular testing beginning in January 2020 for travel-related requests using a gel-based reverse transcriptase PCR (RT-PCR) assay (COVID-19-specific and pan-coronavirus) (4). From February 2020 onward, laboratory-developed real-time RT-PCR assays targeting the SARS-CoV-2 envelope (E) and/or RNA-dependent-RNA-polymerase (RdRp) were implemented, followed by a laboratory-developed E/RdRp/MS2 multiplex assay or Seegene (2019-nCoV assay, Seegene), Xpert (Xpress SARS-CoV-2, Cepheid), Aptima (SARS-CoV-2 assay, Hologic), BD Max (BioGX SARS-CoV-2, BD Molecular Diagnostics), Simplexa (COVID-19 direct test, DiaSorin), or cobas 6800 (SARS-CoV-2 test, Roche) testing (depending on laboratory testing location) for all symptomatic patients (27, 28). Between 29 May 2020 and 4 November 2020, broad asymptomatic testing was made available to all Albertans.

### SARS-CoV-2 antibody testing.

All samples were tested using an orthogonal testing algorithm. Samples were screened by the Abbott Architect SARS-CoV-2 IgG antibody assay, which detects SARS-CoV-2 antibodies directed against the viral nucleocapsid protein. All positives were confirmed by the DiaSorin SARS-CoV-2 IgG antibody assay which detects antibodies directed against the S1 and S2 regions of the spike protein as validated previously in the laboratory (14). Samples that were positive by the screening assay but negative by the confirmation assay were considered negative.

### Data and analysis.

Serology test results were matched by specimen ID numbers to patients’ demographic information, including sex, date of birth, postal code of residence, and sample collection date. Survey periods were limited to the following specimen collection date ranges: 1 to 5 June, 6 to 10 July, 4 to 11 August, 7 to 11 September, 5 to 9 October, 9 to 13 November, and 7 to 10 December 2020 and 11 to 14 January 2021. Results from duplicate specimens of the same survey period were excluded. The rollout of COVID-19 vaccination to health care workers and long-term care residents in Alberta began in December 2020; any patients who had received a vaccine prior to specimen collection were excluded from the analysis.

Crude seropositivity rates were calculated using the total number of samples positive for SARS-CoV-2 antibodies divided by the total number of samples submitted. IgG positivity rates were standardized to 2020 Alberta population estimates by sex and 5-year age groups from Statistics Canada (29). Ninety-five percent confidence intervals (95% CI) were calculated for each positivity rate using Wilson score interval for binomial proportions.

Postal codes were used to classify patients’ residence as the following. (i) Urban: mid-to-major cities (Calgary, Edmonton, Red Deer, Lethbridge, Medicine Hat, Grande Prairie, and Brooks); (ii) Alberta, other: other smaller municipalities; and (iii) rural: any postal code beginning with T0. Postal codes were also matched to patients’ census dissemination areas (DAs), which are geographic units that generally cover a residential population of 400 to 700, allowing serology results to be linked to neighborhood-level socioeconomic statistics from the 2016 Canadian census long-form questionnaire, which was conducted by Statistics Canada (30). These variables included median household income, average household size, marital status by proportions of married/common-law or single Albertans, proportions of highest education level achieved (below high school, high school, diploma/certificate, Bachelor’s degree, or graduate/professional degree), proportions by immigration status (immigrant, recent immigrant within the past 5 years, or nonpermanent resident), visible minority status (31), and group proportions (self-identify as Aboriginal or of African, Chinese, non-Chinese East Asian, South Asian, Latin American, or Middle Eastern descent). The population means of these summary measures were compared between the IgG positive and IgG negative groups using *t* tests.

Finally, previous SARS-CoV-2 nucleic acid testing (NAAT) results extracted from the ProvLab Laboratory Information System were merged with IgG results by patients’ Alberta personal health numbers (PHNs). Patients were then classified as (i) having no NAAT results at any time prior to phlebotomy for the serology tested specimen, (ii) having at least one positive NAAT result prior to serology testing, or (iii) having only negative or inconclusive NAAT results prior to serology testing. Only NAAT testing done prior to the date of the patient’s IgG sample was included in our analysis.

### COVID-19-related public health measures.

Information regarding public health measures instituted, including requirements for isolation, testing, mask mandates, and different stages of loosening of restrictions, are summarized (Table S1) (32). Nonmedical masking was recommended beginning on 6 April 2020 for individuals (>2 years of age) in public spaces who could not maintain a 6-foot distance from others not in their household.
