# Supplementary material for: Pre-Vaccine Positivity of SARS-CoV-2 Antibodies in Alberta, Canada during the First Two Waves of the COVID-19 Pandemic
Source: Microbiol Spectr. 2021 Aug 18;9(1):10.1128/spectrum.00291-21. doi: 10.1128/spectrum.00291-21 (PMC8552659; doi:10.1128/spectrum.00291-21)
Supplement: SUPPLEMENTAL FILE 1 — Supplemental material. Download SPECTRUM00291-21_Supp_1_seq6.pdf, PDF file, 0.6 MB [file spectrum00291-21_supp_1_seq6.pdf]

Supplementary material – TABLE OF CONTENTS

Manuscript title:

Pre-vaccine positivity of SARS-CoV-2 antibodies in Alberta, Canada during the first two waves of the COVID-19 pandemic

Figures

Figure S1. IgG Positivity of major Alberta cities or geographical areas.

Figure S2. A) Crude and B) Adjusted seroprevalence rates according to different criteria for positivity: instrument response ≥ 0.7 (grey dashed line), instrument response ≥ 1.4 (black dashed line), instrument response ≥ 1.4 with confirmation by DiaSorin (DS) (solid black line).

Tables

Table S1. Timeline of COVID-19-associated public health measures implemented in Alberta, Canada.

Table S2. COVID-19 Seroprevalence of Alberta by age group and sex at each monthly snapshot (*N* = 93,993).

Table S3. COVID-19 Seroprevalence by cities or areas of residence at each monthly snapshot, unknown and out of province postal codes excluded (*N* = 92,751).

Table S4. Socioeconomic characteristics of IgG negative and positive patients' neighbourhoods of residence from the Alberta Serosurvey, June 2020 – January 2021.

Table S5. Count of Albertans from the Serosurveys according to their NAAT and IgG results with different criteria (*N* = 66,171).

Table S6. Select demographic distributions of seropositive patients (N = 1237) according to their prior status by NAAT.

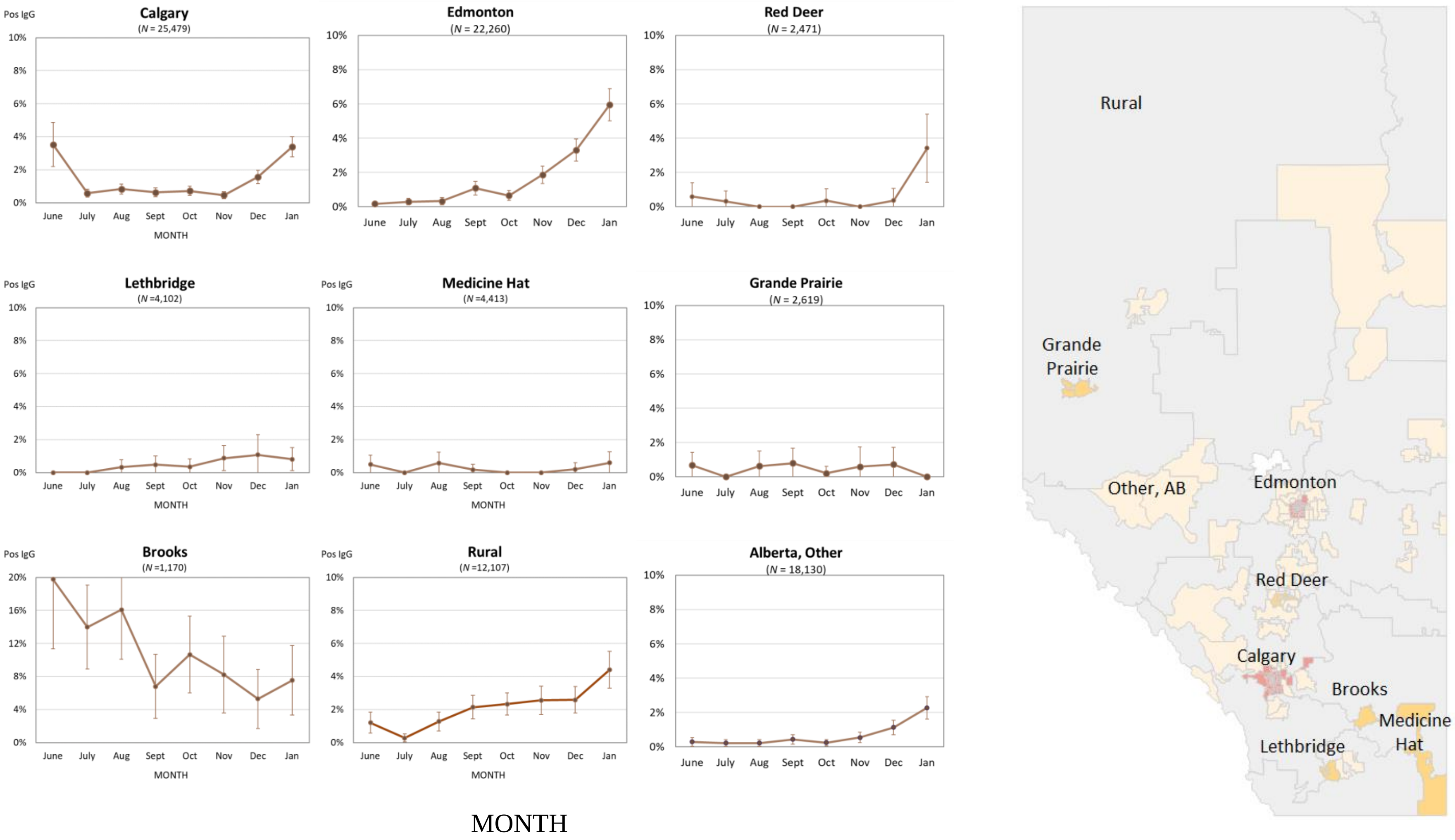

Figure S1. IgG Positivity of major Alberta cities or geographical areas, with 95% CI error bars. Please note the two-times larger y-axis scale for Brooks. Calgary and Edmonton are shaded in red; Grande Prairie, Lethbridge, Medicine Hat, Red Deer and Brooks are shaded in orange; Rural areas shaded in grey; and the remaining cities in the Alberta are shaded in beige.

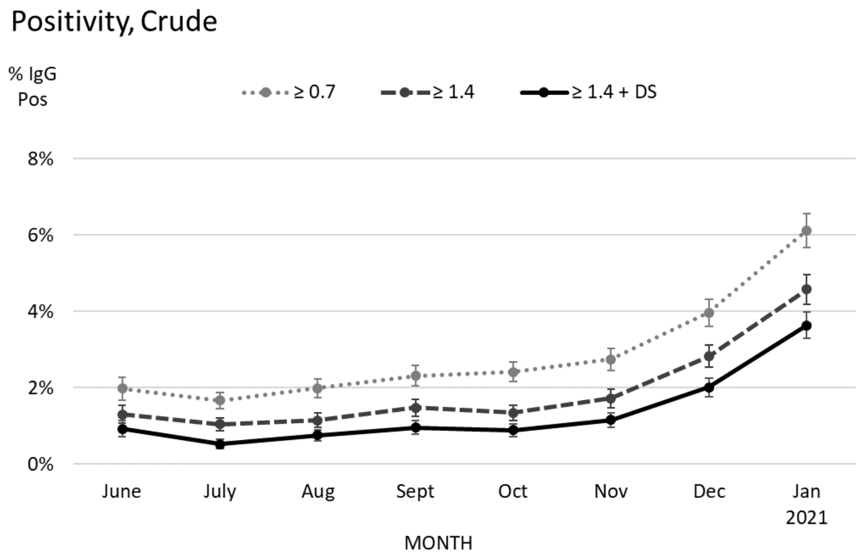

A)

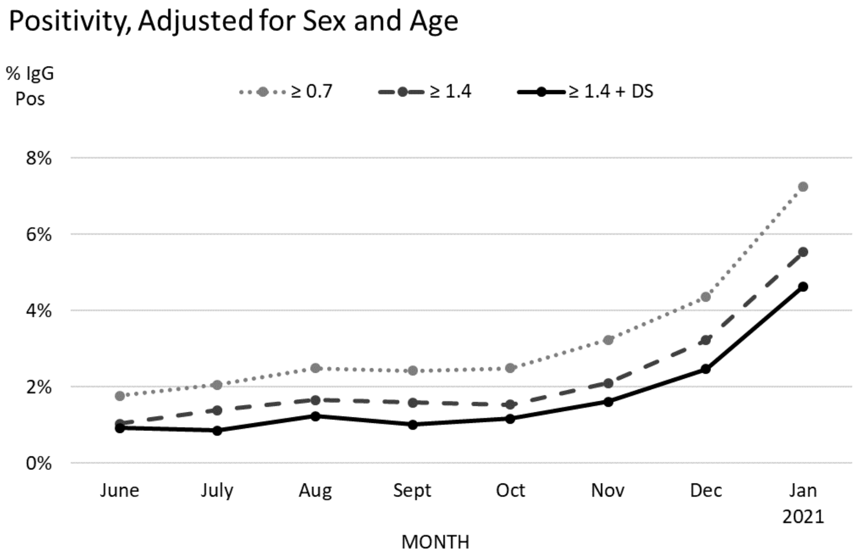

B)

Figure S2. A) Crude and B) Adjusted seroprevalence rates according to different criteria for positivity: instrument response ≥ 0.7 (grey dashed line), instrument response ≥ 1.4 (black dashed line), instrument response ≥ 1.4 with confirmation by DiaSorin (DS) (solid black line).

Table S1. Timeline of COVID-19-associated public health measures implemented in Alberta, Canada.

Date	Measure
March 15, 2020	Province of Alberta declares a public health state of emergency with regards to COVID-19, closing most non-essential businesses, services as well as schools.
April 6, 2020	Use of non-medical face masks in those >2 years of age is advised by public health when indoor spacing of 6 feet (2 meters) or more cannot be maintained.
June 12, 2020	While public health restrictions exist, province permits businesses and in-restaurant dining to resume.
September 1, 2020	Students return to school with a compulsory masking rule in-effect.
October 26, 2020	Government imposes mandatory 15-person limit on social gatherings as cases rise.
November 24, 2020	New public health restrictions by government in response to rising case numbers closing schools, limiting attendance at places of worship, and banning social gatherings.
December 15, 2020	COVID-19 vaccines launched in Alberta for long-term care residents.
January 8, 2021	COVID-19 vaccines made available to front-line healthcare workers managing COVID-19 patients.
January 11, 2021	Students return to in-person learning in schools.
January 14, 2021	Restrictions eased for outdoor gatherings and personal service businesses permitted to open as case counts decline.

Reference: 12.

Table S2. COVID-19 Seroprevalence of Alberta by age group and sex at each monthly snapshot (N = 93,993).

	June			July			August		
Demographic	Survey size n	IgG Positive count n_{POS}	% IgG Positive (95% CI)	Survey size n	IgG Positive count n_{POS}	% IgG Positive (95% CI)	Survey size n	IgG Positive count n_{POS}	% IgG Positive (95% CI)
Overall, crude	8,442	78	0.924 (0.720 – 1.128)	13,175	68	0.516 (0.394 – 0.639)	12,284	92	0.749 (0.596 – 0.901)
Overall, adjusted			0.920 (0.911 – 0.929)			0.859 (0.851 – 0.868)			1.235 (1.225 – 1.246)
Sex									
Male	3,707	39	1.052 (0.724 – 1.381)	5,749	34	0.591 (0.393 – 0.790)	5,214	40	0.767 (0.530 – 1.004)
Female	4,735	39	0.824 (0.566 – 1.081)	7,426	34	0.458 (0.304 – 0.611)	7,070	52	0.736 (0.536 – 0.935)
Unknown									
Age group (y)									
0-9	96	0	0 (0.000 – 0.000)	139	2	1.550 (0.000 – 3.682)	121	4	3.306 (0.120 – 6.491)
10-19	245	2	0.816 (0.000 – 1.943)	449	4	0.891 (0.022 – 1.760)	390	2	0.513 (0.000 – 1.222)
20-29	696	6	0.862 (0.175 – 1.549)	976	11	1.127 (0.465 – 1.789)	946	9	0.951 (0.333 – 1.570)
30-39	1,073	16	1.491 (0.766 – 2.216)	1,687	11	0.652 (0.268 – 1.036)	1,577	13	0.824 (0.378 – 1.271)
40-49	977	15	1.535 (0.764 – 2.306)	1,669	15	0.899 (0.446 – 1.352)	1,478	15	1.015 (0.504 – 1.526)

50-59	1,347	11	0.817 (0.336 – 1.297)	2,140	13	0.607 (0.278 – 0.937)	1,959	22	1.123 (0.656 – 1.590)
60-69	1,706	9	0.528 (0.184 – 0.871)	2,716	5	0.184 (0.023 – 0.345)	2,523	10	0.396 (0.151 – 0.642)
70-79	1,405	11	0.783 (0.322 – 1.244)	2,157	6	0.278 (0.056 – 0.500)	1,979	8	0.404 (0.125 – 0.684)
≥ 80	897	8	0.892 (0.277 – 1.507)	1,252	1	0.080 (0.000 – 0.236)	1,311	9	0.686 (0.240 – 1.133)
Median Age (years, IQR)			48.0 (37.0 – 67.0)			41.5 (32.0 – 55.8)			51.0 (37.3 – 62.8)

Table S2 (continued). COVID-19 Seroprevalence of Alberta by age group and sex at each monthly snapshot (N = 93,993).

	September			October			November		
Demographic	Survey size n	IgG Positive count n_{POS}	% IgG Positive (95% CI)	Survey size n	IgG Positive count n_{POS}	% IgG Positive (95% CI)	Survey size n	IgG Positive count n_{POS}	% IgG Positive (95% CI)
Overall, crude	12,006	115	0.958 (0.784 – 1.132)	13,490	119	0.882 (0.724 – 1.040)	11,471	132	1.151 (0.956 – 1.346)
Overall, adjusted			1.015 (1.005 – 1.024)			1.162 (1.152 – 1.172)			1.607 (1.595 – 1.619)
Sex									
Male	5,149	50	0.971 (0.703 – 1.239)	5,860	46	0.785 (0.559 – 1.011)	5,054	68	1.345 (1.028 – 1.663)
Female	6,857	65	0.948 (0.719 – 1.177)	7,631	73	0.957 (0.738 – 1.175)	6,416	64	0.998 (0.754 – 1.241)
Unknown							1	0	0 (0.000 – 0.000)
Age group (y)									
0-9	86	1	1.163 (0.000 – 3.429)	130	3	2.308 (0.000 – 4.889)	107	4	3.738 (0.144 – 7.333)
10-19	285	3	1.053 (0.000 – 2.237)	377	5	1.326 (0.171 – 2.481)	337	5	1.484 (0.193 – 2.774)
20-29	865	11	1.272 (0.525 – 2.018)	1,029	11	1.069 (0.441 – 1.697)	895	18	2.011 (1.091 – 2.931)
30-39	1,482	13	0.877 (0.402 – 1.352)	1,784	17	0.953 (0.502 – 1.404)	1,567	17	1.085 (0.572 – 1.598)
40-49	1,472	15	1.019 (0.506 – 1.532)	1,769	18	1.018 (0.550 – 1.485)	1,574	24	1.525 (0.919 – 2.130)

50-59	1,898	19	1.001<br>(0.553 – 1.449)	2,249	33	1.467<br>(0.970 – 1.964)	1,879	20	1.064<br>(0.600 – 1.528)
60-69	2,552	26	1.019<br>(0.629 – 1.408)	2,839	18	0.634<br>(0.342 – 0.926)	2,383	22	0.923<br>(0.539 – 1.307)
70-79	2,027	14	0.691<br>(0.330 – 1.051)	2,128	8	0.376<br>(0.116 – 0.636)	1,729	12	0.694<br>(0.303 – 1.085)
≥ 80	1,339	13	0.971<br>(0.446 – 1.496)	1,186	6	0.506<br>(0.102 – 0.910)	1,000	10	1.000<br>(0.383 – 1.617)
Median Age<br>(years, IQR)			58.0<br>(51.5 – 69.0)			52.0<br>(38.0 – 59.3)			48.0<br>(34.0 – 66.0)

Table S2 (continued). COVID-19 Seroprevalence of Alberta by age group and sex at each monthly snapshot (*N* = 93,993).

	December			January 2021		
Demographic	Survey size *n*	IgG Positive count n_{POS}	% IgG Positive (95% CI)	Survey size *n*	IgG Positive count n_{POS}	% IgG Positive (95% CI)
Overall, crude	12,076	241	1.996 (1.746 - 2.245)	11,015	400	3.620 (3.272 - 3.969)
Overall, adjusted			2.462 (2.447 – 2.476)			4.622 (4.602 – 4.641)
Sex						
Male	5,418	123	2.270 (1.874 - 2.667)	4,984	172	3.451 (2.944 - 3.958)
Female	6,657	118	1.773 (1.456 - 2.090)	6,066	228	3.759 (3.280 - 4.237)
Unknown	1	0	0 (0.000 – 0.000)	0	-	-
Age group (y)						
0-9	119	3	2.521 (0.000 - 5.338)	97	7	7.216 (2.067 - 12.366)
10-19	374	7	1.872 (0.498 - 3.245)	329	16	4.863 (2.539 - 7.187)
20-29	917	31	3.381 (2.211 - 4.550)	909	43	4.730 (3.350 - 6.111)
30-39	1655	55	3.323 (2.459 - 4.187)	1480	75	5.068 (3.950 - 6.185)
40-49	1663	36	2.165 (1.465 - 2.864)	1559	64	4.105 (3.120 - 5.090)

50-59	2009	43	2.140 (1.508 - 2.773)	1859	70	3.765 (2.900 - 4.631)
60-69	2519	37	1.469 (0.999 - 1.939)	2301	73	3.173 (2.456 - 3.889)
70-79	1761	21	1.193 (0.686 - 1.699)	1614	37	2.292 (1.562 - 3.023)
≥ 80	1059	8	0.755 (0.234 - 1.277)	901	15	1.665 (0.829 - 2.501)
Median Age (years, IQR)			46.0 (34.0 – 60.0)			48.0 (34.0 – 63.0)

Table S3. COVID-19 Seroprevalence by cities or areas of residence at each monthly snapshot, unknown and out of province postal codes excluded (*N* = 92,751).

	June			July			August		
City/Area	Survey size *n*	IgG Positive count n_{POS}	% IgG Positive (95% CI)	Survey size *n*	IgG Positive count n_{POS}	% IgG Positive (95% CI)	Survey size *n*	IgG Positive count n_{POS}	% IgG Positive (95% CI)
Calgary	737	26	3.528 (2.196 – 4.860)	3,795	22	0.580 (0.338 – 0.821)	3,362	28	0.833 (0.526 – 1.140)
Edmonton	2,988	5	0.167 (0.021 – 0.314)	2,768	8	0.289 (0.089 – 0.489)	2,833	9	0.318 (0.110 – 0.525)
Red Deer	341	2	0.587 (0.000 – 1.397)	326	1	0.307 (0.000 – 0.907)	375	0	0 (0.000 – 0.000)
Lethbridge	198	0	0 (0.000 – 0.000)	601	0	0 (0.000 – 0.000)	625	2	0.320 (0.000 – 0.763)
Medicine Hat	607	3	0.494 (0.000 – 1.052)	594	0	0 (0.000 – 0.000)	521	3	0.576 (0.000 – 1.226)
Grande Prairie	448	3	0.670 (0.000 – 1.425)	445	0	0 (0.000 – 0.000)	318	2	0.629 (0.000 – 1.498)
Brooks	86	17	19.77 (11.35 – 28.18)	179	25	13.97 (8.888 – 19.05)	143	23	16.08 (10.06 – 22.11)
Rural	1,164	14	1.203 (0.577 – 1.829)	1,826	5	0.274 (0.034 – 0.514)	1,484	19	1.280 (0.708 – 1.852)
Alberta, Other	1,806	8	0.443 (0.137 - 0.749)	2,577	6	0.233 (0.047 - 0.419)	2,305	5	0.217 (0.027 - 0.407)

Table S3 (continued). COVID-19 Seroprevalence by cities or areas of residence at each monthly snapshot, unknown and out of province postal codes excluded (*N* = 92,751).

	September			October			November		
City/Area	Survey size *n*	IgG Positive count n_{POS}	% IgG Positive (95% CI)	Survey size *n*	IgG Positive count n_{POS}	% IgG Positive (95% CI)	Survey size *n*	IgG Positive count n_{POS}	% IgG Positive (95% CI)
Calgary	3,626	23	0.634 (0.376 – 0.893)	3,603	26	0.722 (0.445 – 0.998)	3,481	16	0.460 (0.235 – 0.684)
Edmonton	2,508	27	1.077 (0.673 – 1.480)	3,083	20	0.649 (0.365 – 0.932)	2,737	51	1.863 (1.357 – 2.370)
Red Deer	244	0	0 (0.000 – 0.000)	286	1	0.350 (0.000 – 1.034)	298	0	0 (0.000 – 0.000)
Lethbridge	632	3	0.475 (0.000 – 1.011)	571	2	0.350 (0.000 – 0.835)	576	5	0.868 (0.110 – 1.626)
Medicine Hat	600	1	0.167 (0.000 – 0.493)	658	0	0 (0.000 – 0.000)	426	0	0 (0.000 – 0.000)
Grande Prairie	381	3	0.787 (0.000 – 1.675)	480	1	0.208 (0.000 – 0.616)	169	1	0.592 (0.000 – 1.748)
Brooks	162	11	6.790 (2.916 – 10.66)	169	18	10.65 (6.000 – 15.30)	134	11	8.209 (3.561 – 12.86)
Rural	1,591	34	2.137 (1.426 – 2.848)	1,928	45	2.334 (1.660 – 3.008)	1,291	33	2.556 (1.695 – 3.417)
Alberta, Other	2,113	9	0.426 (0.148 - 0.704)	2,571	6	0.233 (0.047 – 0.420)	2,212	12	0.542 (0.236 - 0.849)

Table S3 (continued). COVID-19 Seroprevalence by cities or areas of residence at each monthly snapshot, unknown and out of province postal codes excluded (*N* = 92,751).

	December			January 2021		
City/Area	Survey size n	IgG Positive count n_{POS}	% IgG Positive (95% CI)	Survey size n	IgG Positive count n_{POS}	% IgG Positive (95% CI)
Calgary	3,511	55	1.567 (1.156 – 1.977)	3,364	114	3.389 (2.777 - 4.000)
Edmonton	2,904	96	3.306 (2.656 – 3.956)	2,439	145	5.945 (5.006 - 6.884)
Red Deer	279	1	0.358 (0.000 – 1.060)	322	11	3.416 (1.432 - 5.400)
Lethbridge	278	3	1.079 (0.000 – 2.294)	621	5	0.805 (0.102 - 1.508)
Medicine Hat	504	1	0.198 (0.000 – 0.587)	503	3	0.596 (0.000 - 1.269)
Grande Prairie	277	2	0.722 (0.000 – 1.719)	101	0	0.000 (0.000 - 0.000)
Brooks	151	8	5.298 (1.725 – 8.871)	146	11	7.534 (3.253 - 11.82)
Rural	1,506	39	2.590 (1.787 – 3.392)	1,317	58	4.404 (3.296 - 5.512)
Alberta, Other	2,474	27	1.091 (0.682 - 1.501)	2,072	47	2.268 (1.627 - 2.909)

Table S4. Socioeconomic characteristics of IgG negative and positive patients' neighborhoods of residence from the Alberta Serosurvey, June 2020 – January 2021.[a]

	IgG NEGATIVE		IgG POSITIVE		p NEG-POS[c]
Category	Mean[b]	95% CI	Mean[b]	95% CI	
Median Household Income (CAD)	97,500	97,300 - 97,800	87,500	85,600 - 89,500	< 0.001
Married/Common-Law (ref: Single, %)	59.2	59.1 - 59.2	58.1	57.5 - 58.7	< 0.001
Average Household Size	2.61	2.61 - 2.62	2.72	2.69 - 2.76	< 0.001
Highest Level of Education					
Below High School (%)	17.5	17.4 - 17.5	21.0	20.4 - 21.7	< 0.001
High School (%)	28.2	28.1 - 28.2	28.9	28.5 - 29.3	< 0.001
Diploma/Certificate (%)	31.8	31.7 - 31.8	29.9	29.4 - 30.3	< 0.001
Bachelor's (%)	22.5	22.5 - 22.6	20.1	19.4 - 20.9	< 0.001
Graduate/Prof. Degree (%)	5.40	5.37 - 5.44	4.86	4.58 - 5.13	< 0.001
Immigration Status					
Immigrant (%)	21.2	21.1 - 21.3	27.2	26.3 - 28.2	< 0.001
Immigrant Last 5 yrs (%)	5.13	5.10 - 5.17	8.23	7.78 - 8.68	< 0.001
Non-Permanent Resident (%)	1.76	1.74 - 1.78	2.13	1.93 - 2.33	< 0.001
Visible Minority Group					
Any Visible Minority (%)	22.9	22.7 - 23.0	30.9	29.5 - 32.4	< 0.001
Aboriginal (%)	5.61	5.56 - 5.66	6.42	5.67 - 7.17	0.034
African (%)	3.17	3.14 - 3.20	5.81	5.33 - 6.30	< 0.001
Chinese (%)	3.86	3.82 - 3.90	3.63	3.31 - 3.95	0.157
East Asian (Excl. Chinese) (%)	6.01	5.97 - 6.05	7.66	7.26 - 8.06	< 0.001
Latin American (%)	5.66	5.59 - 5.73	8.48	7.63 - 9.32	< 0.001

Middle Eastern (%)	1.38	1.37 - 1.39	1.75	1.61 - 1.89	< 0.001
South Asian (%)	1.85	1.82 - 1.87	2.50	2.27 - 2.74	< 0.001

[a] Unknown address and out of province residents excluded; n = 92,758; n_{POS} = 1,217

[b] Mean figures for Serosurvey patients' neighbourhoods calculated from census-derived statistics of their Dissemination Areas (DAs) of residence.

[c] p-value calculated from t-test comparing IgG Negative and Positive patients.

Table S5. Count of Albertans from the Serosurveys according to their NAAT and IgG results with different criteria (N = 66,171)[a]

	IgG Result (≥1.4RU+DS)		IgG Result (≥1.4 RU)		IgG Result (≥0.7 RU)	
NAAT Testing Status/Result	Positive (%)	Negative (%)	Positive (%)	Negative (%)	Positive (%)	Negative (%)
No prior NAAT result found	485 (0.73)	65,686 (99.27)	804 (1.22)	65,667 (99.23)	1400 (2.12)	65,071 (98.33)
Any prior NAAT result						
At least one Positive result	573 (0.87)	294 (0.44)	662 (1.00)	207 (0.31)	739 (1.12)	130 (0.20)
Only Negative/Inconclusive results	179 (0.27)	25,665 (38.79)	309 (0.47)	25,538 (38.59)	539 (0.81)	25,308 (38.25)
Any NAAT result > 2 weeks prior						
At least one Positive result	551 (0.83)	214 (0.32)	618 (0.93)	147 (0.22)	690 (1.04)	75 (0.11)
Only Negative/Inconclusive results	180 (0.27)	23,259 (35.15)	302 (0.46)	23,137 (34.97)	516 (0.78)	22,923 (34.64)

[a] Excluding Albertans with no recorded personal healthcare numbers.

Table S6. Select demographic distributions of seropositive patients (N = 1237) according to their prior status by NAAT.

	Positive NAAT		Negative or no NAAT		p^a
Demographic Category	%	95% CI	%	95% CI	
Sex					
Male	44.3	40.3 - 48.4	47.1	43.3 - 50.9	0.323
Female	55.7	51.6 - 59.7	52.9	49.1 - 56.7	0.323
Age group (y)					
0-9	0.7	0.1 - 1.3	2.9	1.6 - 4.1	0.005
10-19	2.8	1.5 - 4.0	4.2	2.7 - 5.7	0.177
20-29	9.4	7.2 - 11.6	13.0	10.4 - 15.5	0.051
30-39	15.7	12.9 - 18.5	18.7	15.7 - 21.6	0.169
40-49	17.5	14.6 - 20.3	15.1	12.3 - 17.8	0.255
50-59	19.4	16.4 - 22.4	18.1	15.1 - 21.0	0.559
60-69	16.1	13.3 - 18.8	16.0	13.2 - 18.7	0.965
70-79	9.4	7.2 - 11.6	9.5	7.3 - 11.7	0.970
≥ 80	9.1	6.9 - 11.3	2.7	1.5 - 3.9	< 0.001
Geography					
Urban, Major – Calgary	28.7	25.0 - 32.4	22.1	18.9 - 25.3	0.009
Urban, Major – Edmonton	33.0	29.2 - 36.9	26.6	23.2 - 30.0	0.014
Urban, Midsize	26.2	22.6 - 29.8	23.7	20.4 - 27.0	0.306
Rural	12.1	9.4 - 14.7	27.6	24.1 - 31.0	< 0.001
Median Household Income (CAD)	92,000	89,000 – 94,900	83,600	81,300 - 86,300	< 0.001

[a]p-value calculated using chi-square tests for sex and age variables; using t-tests for geography and household income variables.
